# Italian Physicians’ Opinions on Rotavirus Vaccine Implementation

**DOI:** 10.3390/pathogens6040056

**Published:** 2017-11-03

**Authors:** Valentin Mita, Michele Arigliani, Laura Zaratti, Raffaele Arigliani, Elisabetta Franco

**Affiliations:** 1 Department of Biomedicine and Prevention, University of Rome Tor Vergata, 00133 Rome, Italy; valentinmita@libero.it (V.M.); laura.zaratti@uniroma2.it (L.Z.); 2 Department of Clinical and Experimental Medical Sciences, University Hospital of Udine, 33100 Udine, Italy; michelearigliani@gmail.com; 3 Department of Pediatrics, Università Politecnica delle Marche, 60126 Ancona, Italy; raffaelearigliani@gmail.com

**Keywords:** rotavirus, new vaccines, immunization, coverage, physicians, knowledge, attitudes, beliefs, intentions

## Abstract

Rotavirus (RV) infection is the main cause of severe acute gastroenteritis (GE) in the pediatric population and has a major impact in both developing and industrialized countries. The reduction of severe RVGE cases, followed by death or hospitalization, is considered the main benefit of RV vaccination, even though its implementation often faces obstacles. In Italy, the recently approved National Immunization Plan aims to overcome the differences among regions, offering a universal free RV vaccination. The aim of the study was to evaluate the opinions on benefit and acceptability of RV vaccination related to the perception of the burden of RV disease. Data were collected from 108 physicians in 2015 by a questionnaire consisting of 12 questions; some answers were compared with those obtained with a similar tool in 2011. The majority of respondents (76.2%) was convinced of the benefit of the vaccine and 57.4% recommended it routinely, but more than half indicated a <25% adherence to RV vaccination among their patients. As the main reasons of vaccine refusal, skepticism about the vaccine (60.4%) and its cost (34.1%) were indicated. Our data confirm that more information and counselling are needed to increase RV vaccine coverage.

## 1. Introduction

Rotavirus (RV) infection is the leading cause of severe acute gastroenteritis in the pediatric population worldwide. According to the latest estimates, among children under 5 years old (<5 years) there are about 215,000 (range 197,000–233,000) deaths annually due to RV gastroenteritis (RVGE). The highest mortality rates are in developing countries of sub-Saharian Africa and South Asia [1]. Although the infection is characterized by ubiquity and universality for the early childhood period, multiple factors present in countries with different levels of development result in substantial differences in the burden of disease [2]. 

Despite very low mortality rates (up to 1/100,000 <5 years), industrialized countries are characterized by a significant economic and psychosocial impact of this disease [3,4,5]. In Italy, RVGE has an estimated incidence of about 5/100 children <5 years [6], which was reported to result annually in 320,000 cases handled at home, 80,000 visits to the physician’s office, 10,000 hospitalizations, and 11 deaths [7]. A recent study found an average annual hospitalization rate for RVGE of 296/100,000 children <5 years, with 81% of cases in the <3 years age group [8]. The burden of disease is believed to be underestimated by up to 50% [8,9,10,11] and results in an economic impact of 144 million euros in health and social costs, as well as in overstretching and cluttering emergency departments (ED) and primary healthcare facilities [7].

Vaccination is considered as the most promising public health strategy for reducing the burden of disease. Since 2009, the World Health Organization (WHO) and other authoritative scientific societies recommend the introduction of RV vaccination in all National Immunization Programs (NIPs), regardless of a country’s level of development [2,12].

Two live, attenuated, second-generation RV vaccines were authorized in 2006 [13], and can be administered in two (RV1) or three (RV5) doses any time from the age of 6 weeks, with an interval of at least four weeks between the doses [2,12,14]. In 2009, WHO were established the age limits for the start of RV vaccination [2] to reduce the risk of intussusception (a rare occurrence in newborns, but whose incidence increases up to 10 times in the period from 3 to 12 weeks after birth [14,15]. These recommendations were revised in 2012 because they could negatively affect vaccine coverage rates in countries where vaccination is often delayed and the risk of death due to RV infection is high [12]. In industrialized countries, the risk of intussusception may increase the vaccine hesitancy and the latest recommendations from the European Society for Pediatric Infectious Diseases suggest the administration of the first dose of the RV vaccine at the age of 6 to 8 weeks after birth [16]. However, the recommendation to begin vaccination series not before the sixth week remains valid, due to presence of passive maternal origin protection that may interfere with immune response to vaccine [2,12,14,16]. Unlike many vaccinations that may be started at any age, a delayed RV vaccine administration determines the decrease of its effectiveness due to the acquisition of natural immunity, which is the reason why there is no vaccine recovery [17,18].

Many studies from countries that have adopted universal RV vaccination showed a considerable impact in preventing serious forms of disease and its complications as well as a decrease in the number of hospitalizations and visits to primary care in the first years of implementation [19,20]. Similar, but less pronounced effects were obtained in countries with moderate (20–40%) vaccine coverage [21]. In Italy (Sicily Region), three years after the introduction of universal RV vaccination with an average vaccine coverage of 37%, RVGE hospitalization rates fell by 31.5% overall and 41% in the 0–35 months age group, with more pronounced results in areas with vaccine coverage >45%. The same authors noted an epidemiological shift towards the spring months, resulting in lightening the demand for pediatric health care facilities during the peak season shared by RSV infection and flu [22,23]. In addition, due to the effect of herd immunity, costs could be cut further for illness management among older children and adults (mostly family members) who are not subject to vaccination for their age [17,24,25].

As of July 2017, 92 countries have introduced the RV vaccine into their National Immunization Program, seven of these at the subnational level, and other 26 countries have declared their intention to make this introduction. These data include 19 WHO European Region countries, notably with only Sweden and Italy (until January 2017) having joined RV vaccine introduction at the regional level [26]. In Italy, following a long, gradual, and heterogeneous pathway, “with a leopard patch” and with different forms of supply [6,27], the National Plan for Prevention and Vaccination (PNPV) 2017–2019 was approved. The PNPV forecasts the overcoming of differences between the regions through the active and free offer of RV1or RV5 throughout the national territory [28,29]. However, the RV vaccine, although strongly recommended, is not part of the 10 recently made mandatory vaccines [30].

In the context of limited economic resources, the inclusion of new vaccines (e.g., the RV vaccine) in public immunization programs is conditioned not only by factors such as severity and burden of disease, and efficacy and safety of available vaccines, but also by their efficiency, which in turn is conditioned by acceptability and priority versus other public health interventions [31,32]. In Italy, where health policy management is the task of the regions, decision-makers are often faced with difficult issues to deal with, especially when primary care practitioners have diverging or even contradictory opinions. Their knowledge, attitudes, and beliefs about vaccines significantly affect the outcomes of the whole intervention, and studies from similar backgrounds emphasize that the support of healthcare practitioners should not be taken for granted. In the absence of their enduring and fully shared support, a successful implementation of RV vaccination is almost impossible [21,32,33].

The aim of this study is to determine and evaluate the perception of the burden of RV disease among doctors involved in vaccination practice, their opinions on the RV vaccine usefulness and acceptability, and finally the perceived obstacles and proposals to overcome these in order to identify possible areas of intervention that would facilitate the implementation of the RV vaccination.

## 2. Results

**Figure 1:**
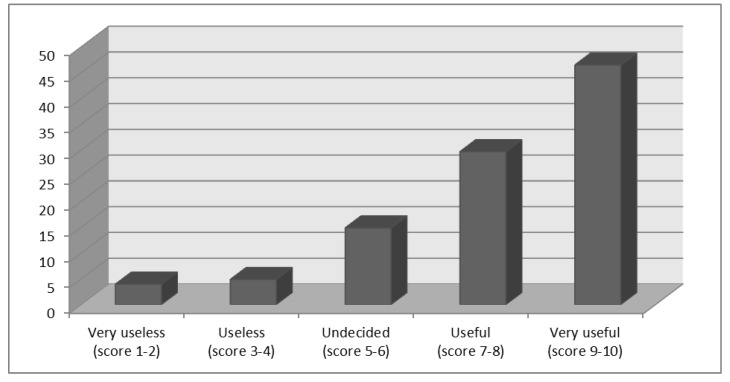
The perception of usefulness of the Rotavirus vaccine (%).

A total of 108 questionnaires were collected in 2015. Most responding physicians (76.2%) consider the RV vaccine useful or very useful (29.7% and 46.5%, respectively), 14.9% were undecided, and others had deemed it useless or very useless (4.9% and 4.0%, respectively) (Figure 1). The mean rating was 7.7 ± 2.3 (median 8.0, range 10–1).

However, only 57.4% of doctor respondents routinely recommended the RV vaccine during their professional activities, and more than half (55.0%) indicated a low (<25%) adherence of their patients to vaccination. The skepticism about the vaccine (60.4%) and its cost (34.1%) were indicated among the main causes of vaccine refusal in households. Other reasons (i.e., logistics difficulties, etc.) had a marginal total weight (5.5%). The majority of physicians believed that the vaccine should be offered free of charge to all (61.3%) or at least to those at risk (30.2%), while 7.5% indicated the co-payment regime as the best option, and only one out of 106 respondents chose the “payment” option. If the rotavirus vaccine was free of charge, the majority of respondents (81.1%) would recommend it to their patients. The “ensure free of charge access to the vaccine” as the most useful strategy to improve its implementation was mentioned in 21.3% of responses, placing it second after the acquisition of major vaccine counselling skills (34.0%).

A proportion of 67.0% of respondents indicated the consultation of scientific literature as the main source of information about the RV vaccine. This was followed by participation at conferences dedicated to the topic, whilst website consultation was selected only in 4.7% of cases. Even in the context of communicating with their patients, 67.0% of interviewed physicians never or rarely (39.6% and 27.4%, respectively) recommended consulting a reliable reference website. In addition, 67.9% of respondents never or rarely (48.1% and 19.8%, respectively) used specifically produced printed promotional materials, preferring to use verbal communication during visits. A rate of 80.6% of those interviewed stated that they would make sure that at the end of communication parents would not have doubts or questions about RV vaccination.

**Figure 2:**
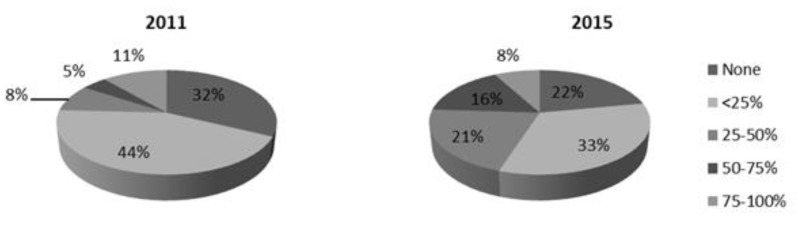
Adherence to Rotavirus vaccination among patients (2011 vs. 2015).

Analyzing the common data of a 2011 survey, we found some differences in the obtained results. For example, there was a significant decrease (*p* < 0.0005) from 75.8 to 55.0% (2011 and 2015, respectively) of physicians reporting a low (<25%) adherence to RV vaccination (Figure 2).

**Figure 3:**
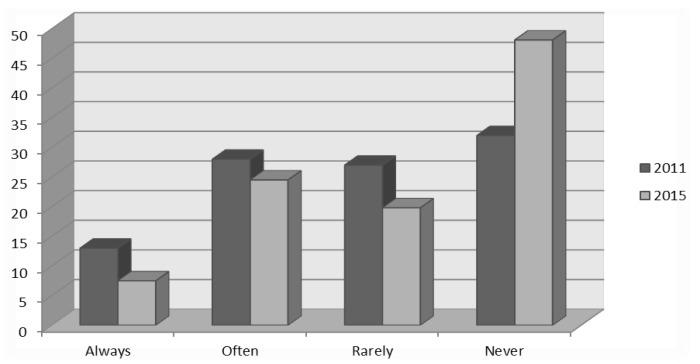
The use of printed promotional materials on the RV vaccine by physicians (2011 vs. 2015).

There was an apparent increase, even if it did not reach statistical significance (0.05 < *p* < 0.1), of the proportion of respondents who would recommend a free of charge vaccine (from 71.8 to 81.1%, compared to 2011). On the other hand, the use of printed promotional materials on the RV vaccine as a startup tool to subsequent communication showed a reverse trend, with an increase from 32.0 to 48.1% (*p* < 0.05) of specialists who never use it in their daily practice (Figure 3). No differences were observed (*p* > 0.05) in monitoring of the end-of-visit effectiveness of communication.

## 3. Discussion and Conclusions

The negative effects of international media clamor on suspected adverse events of vaccines are also present in Italy. Fortunately, vaccine coverage rates remain generally high, indicating that vaccinations still remain a widely accepted public health strategy. Nevertheless, in 2015, for the first time in years, the safety limits were not reached throughout the national territory. As a consequence, measles cases dramatically increased (reaching about 4500 cases recorded since the beginning of 2017) and a strong concern for the control of other vaccine-preventable infectious diseases has been raised [34,35,36,37]. The postponement of implementation for some vaccinations whilst others became mandatory [29,30], along with many other factors, could affect the achievement of objectives set in the new National Immunization Program (PNPV 2017–2019) [28]. Specifically, the RV vaccine, which is not included among the 10 recently made mandatory vaccines, is likely to be more vulnerable in terms of vaccine coverage.

Partly, suboptimal rates of vaccine coverage can be attributed to vaccine hesitancy, which is a complex and widespread phenomenon in different countries regardless of their level of development. The widespread use of any type of information on the web increases doubts and skepticism even in the most experienced parents. Therefore, parental concerns or fears for subsequent vaccinations, especially when it comes to a new vaccine, and parental acceptance depends on numerous socioeconomic and cultural factors, besides cognitive ones [38,39].

Despite the substantial and growing impact of modern media, healthcare workers are still strong supporters of vaccination and the most important source of information on available vaccines, and their opinion often prevails on the personal views of those they assist [38,40,41,42]. According to the annual (2016) Report on the Social Situation in Italy, in the last few years the media (such as television and Internet) has contributed to a radical change in the medical worker-patient relationship. About half (50.9%) of Italians prefer a shared therapeutic choice, based on dialogue with close collaboration with physicians in decision-making about their own health. Furthermore, the crucial role of GPs (including pediatricians) as a benchmark for patients remains as important as ever: even people who are self-informed on the web about their health in 73.3% of the cases indicate GPs as the main source of health information, and about half of Italians attribute to them the responsibility to provide reliable information [43]. In our study, only 57.4% of respondents stated that they routinely recommend the RV vaccine to their patients, and its acceptance by parents, despite some enhancement in 2015, still remains low: more than half of interviewees indicated a less than 25% adherence to RV vaccination. These findings are in agreement with available data coming from countries where this vaccination is not publicly funded, and are lower than instances where the universal principle is applied [21,44,45,46]. In any case, the observed increase in RV vaccination adherence from 2011 to 2015, as well as an apparent increase in the number of physicians who would recommend a free of charge vaccine, seem to be encouraging signals. Some authors report that the positive effects of herd immunity to RV infection have appeared already by the achievement of a 19–25% vaccine coverage [14]. Thus, a little extra effort to promote this vaccination would be desirable, especially in categories with a lower vaccine coverage.

The weak professional and parental interest for the RV vaccine could be partly explained by a low perception of the burden of disease in terms of the severity of clinical forms and mortality which is manifested in a skepticism towards this vaccine, indicated in our study as the leading cause of vaccination refusal (60.4% of responses). The lack of a free of charge vaccine can deeply affect both the physicians’ decision to recommend and the parents’ acceptance of the vaccination. Indeed, 1/3 of interviewees indicated the cost of the vaccine (preceded only by skepticism) as another main cause of refusal in households; 2/3 considered that the vaccine should be offered free of charge to all; and 4/5 expressed the intentions to recommend it if it was free of charge. The study was done when the RV vaccine was not yet included in the NIP (PNPV), hence it was not yet publicly funded on a national scale. Many respondents perceived the non-gratuity of the vaccination as an important obstacle for its successful recommendation. 

Certainly, considering that from the beginning of 2017 this vaccine has been offered free of charge to all newborns in Italy, some concerns could be solved not only in the parents, but also among healthcare workers who may hesitate to recommend a fee for a vaccination. The assumption by the state of all charges for a public health intervention can in itself constitute a major and authoritative recommendation.

We found a drop in the use of printed promotional materials by physicians between 2011 and 2015 and the lack of interest in some modern information tools (e.g., websites) in the promotion of the RV vaccination. In 2011, physicians were not asked whether they indicated reference websites to their patients, but in 2015 less than 35% recommended them. Furthermore, the presence among interviewees of some objectors to the use of the RV vaccine (e.g., 8.9% who deemed this vaccine useless and 18.9% who would not recommend even if it was free of charge) is worrying. In theory, this professional category should be represented, par excellence, by paladins for a strongly recommended and worldwide acknowledged intervention. In addition, poor vaccine coverage represents an alarm signal not only for the quality of knowledge, beliefs, and attitudes of healthcare workers, but also (and above all) for dramatic consequences which may arise due to missed vaccinations [36].

The strength of this study is that we obtained information on RV vaccination from a nationwide, albeit limited, population. In Italy, as in other countries, this vaccination has had and still has difficulties in acceptance, and few data on its implementation are available. Many studies coming from different realities have shown a remarkable and an immediate positive effect of this public health intervention. Unfortunately, it is also true that countries where RV vaccination is successfully implemented are exceptions rather than the rule. Still, present perplexities on its implementation feed the already highly widespread phenomenon of vaccine hesitancy. We assessed motivations that can lead to doubts among physicians involved in vaccination and we managed to gain some ideas for recommendations to overcome some obstacles in the implementation of this vaccine, taking into account the Italian reality. Furthermore, we realize that the presence of perplexities in physicians, directly involved in the information, management, and implementation of the vaccination, may indicate the increasing hesitancy in other healthcare workers and, even more, in the general population. 

This study also has limitations. A convenience sampling was applied, recruiting doctors who were participating in a course about vaccination counselling in order to improve their ability to communicate effectively with parents about vaccines. Their opinions may not reliably represent those of the whole population of Italian physicians working in vaccination centers. Therefore, we cannot draw from this sample any absolute generalizations, but only few tips and suggestions. For this reason, we present only a descriptive analysis, consciously avoiding in-depth analysis data. 

In conclusion, this study showed that Italian doctors working in vaccination centers are generally strong supporters of the RV vaccination, although some of them were hesitant about recommending this vaccine in their routine practice, because of the cost of the vaccine. Vaccination coverage will probably improve now that the vaccine has become free for all newborns, although educational intervention about the RV vaccination for physicians are still needed in order to convince, for example, those interviewees who declared that they did not to trust the utility of the RV vaccine. The next step should be field tests and a well-balanced implementation of interventions and strategies, trying to improve immunization rates [47]. Further prospective studies, applying the methodology proposed in this study but with more extensive and in-depth analysis of the determinants of physicians’ opinion about the RV vaccination, could probably contribute to its implementation and to increase vaccination coverage. 

## 4. Materials and Methods 

The study involved physicians practicing vaccination across the country. Data were collected during residential courses about vaccine counselling undertaken in 2015 in different Italian towns. Prior to the beginning of the course, the participants were provided and encouraged to complete an anonymous cognitive questionnaire consisting of 12 closed-ended questions about the RV vaccination. We compared the results of four questions that were the same but were part of another questionnaire administered in a confrontable setting in 2011. Specifically, the questions concerned adherence to RV vaccination; intention to recommend the vaccine if this was free of charge; use of printed promotional materials, and control of the effectiveness of promotional communication on the vaccine.

The obtained data were entered into an Excel spreadsheet, to be processed using descriptive statistical methods. To determine the statistical significance of differences found in the comparison of common data with the 2011 survey, we performed the chi-square test for contingency tables (Pearson’s chi-square).
